# Effect of Different Finishing Strategies and Steer Temperament on Animal Welfare and Instrumental Meat Tenderness

**DOI:** 10.3390/ani11030859

**Published:** 2021-03-18

**Authors:** Marcia del Campo, Xavier Manteca, Juan Manuel Soares de Lima, Gustavo Brito, Pilar Hernández, Carlos Sañudo, Fabio Montossi

**Affiliations:** 1 INIA Tacuarembó, Ruta 5 Km 386, C.P.45000 Tacuarembó, Uruguay; jsoaresdelima@inia.org.uy (J.M.S.d.L.); gbrito@inia.org.uy (G.B.); fmontossi@inia.org.uy (F.M.); 2 Universidad Autónoma de Barcelona, 08193 Bellaterra, Spain; xavier.manteca@uab.es; 3 Universitat Politècnica de València, Camino de Vera s/n, 46022 Valencia, Spain; phernan@dca.upv.es; 4 Universidad de Zaragoza, Miguel Servet 177, 50013 Zaragoza, Spain; csanudoastiz@gmail.com

**Keywords:** steers, temperament, acute phase proteins, fecal glucocorticoids, meat tenderness

## Abstract

**Simple Summary:**

Animal welfare is one of the most important quality attributes for meat consumers and a potential tool for differentiation in farms where husbandry is based on extensive rearing systems and high animal welfare standards. However, there is not enough contextualized scientific information in relation to these systems and some others that are emerging, with a higher level of intensification. In this experiment, we compared animal welfare between different fattening systems, through the combination of several indicators regarding physiology, behavior, health, productivity, and meat tenderness. Animal temperament, as well as its impact on welfare and meat quality, was also considered. From our results, we concluded that finishing strategies based on pastures that ensure an adequate level of nutrition and health would be more appropriate for fattening animals, from both the animal welfare and the meat quality perspectives. Intensification up to certain levels (pasture plus supplement), without deprivation of certain behaviors and with constant monitoring of health, should provide productive benefits without compromising animal welfare. In confined systems with 9 m^2^ per animal, the challenge is greater in relation to animal welfare because of restrictions of important behaviors and greater risks of dietary diseases. It is considered that, if some conditions of the confined system are improved, such as the space available per animal and the strict prevention and constant monitoring of diet disorders, it could be a valid alternative for fattening cattle. Temperament could be improved through good handling, with positive impacts on welfare, productivity, and meat tenderness.

**Abstract:**

The aim of this experiment was to evaluate the effect of different fattening systems from pasture to concentrate and temperament on animal welfare (AW) and meat quality (MQ). Eighty-four Hereford steers were randomly assigned to the following groups: T1, pasture (4% of animal live weight: LW); T2, pasture (3% LW) plus concentrate (0.6% LW); T3, pasture (3% LW) plus concentrate (1.2% LW); T4, an ad libitum concentrate treatment. Temperament was assessed by three individual tests: crush score, flight time, and exit speed, building a multicriterial temperament index (TIndex). The flight zone was also registered for each treatment. AW was assessed through the integration of indicators of productivity, physiology, and behavior, as well as by monitoring the health status within each treatment. Shear force was registered for MQ. Differences in average daily gain were due to the different energetic composition of the diets (T4 > T3 > T2 > T1) and were not attributable to animal welfare problems. Animals from T4 had the higher average daily gain (ADG) but welfare was negatively affected, being evident through physiological indicators, the restriction or deprivation of relevant behaviors, diet-related diseases, and mortality. T1, T2, and T3 did not appear to compromise animal welfare. However, strict preventive measures and monitoring should be taken during the habituation process and when using any new diet that includes concentrate, because of possible dietary diseases. Shear force values were lower in T1. None of the animals in our experiment were excitable or aggressive, but there was a positive response to handling in all treatments. In addition, regardless of diet, calmer animals had higher average daily gain and lower shear force values; thus, temperament appears to have a significant influence on productivity and meat quality.

## 1. Introduction

Public sensitivity to animal welfare has risen considerably in recent years and is now a strong issue around the world, being increasingly recognized as an important component in the trade of farm animals and their products [1,2]. From the consumer side, there is an increasing concern about the sustainability of the intensification of animal industries and its potential damages on the environment, human health, and animal welfare. It was highlighted by research that carefully informed consumers have a favorable image about extensive livestock systems and associate them with positive attributes about meat, while more intensive systems create negative expectations and may influence and penalize the qualitative assessment of the meat [3]. According to Stampa [4], pasture-raised livestock products represent a premium niche with an extra value through a cleaner environmental footprint and care for animal welfare, including wildlife. The debate is open about the sustainability of the intensification of animal production systems [5], and scientific information is missing. Therefore, information about animal welfare, expressed in terms of rearing conditions, can be a major determinant of animal-based food acceptability, thus providing a potential tool for meat differentiation in traditional farms where husbandry is based on extensive rearing systems and high animal welfare standards. We consider that, on a national level, the welfare of farm animals is one of many factors determining a nation’s reputation in the international community [6]. The traditional livestock-exporting countries mainly associated with beef exports (Argentina, Brazil, Canada, Paraguay, the United States of America, and Uruguay) and those emerging (Chile and Mexico) have incorporated different aspects of animal welfare into their regulations and practices [5,7]. However, those recommendations are based on scientific information generated mainly from intensive production systems. That is why contextualized, regional and national scientific information is needed [8]. Uruguayan meat is mainly based on rangeland pastures, where the use of hormones and animal protein in feeds is prohibited by law since 1978 and 1996, respectively, as is the use of antibiotics as growth promoters. During the last few years, due to international meat prices and the opening of new markets, intensive fattening systems increased, including a wide range of feeding alternatives between pasture and/or concentrate utilization. The use of feedlots (outdoor) is very restricted but is increasing. This could result in differences in terms of animal welfare, as well as carcass and meat quality, which require to be studied. In this context and as a meat exporter country, it is compulsory for Uruguay not only to provide good-quality and safe meat to the world, but also to have and project a sustainable and welfare-friendly image [8]. The challenge is to produce a fattening strategy to improve the final product, without modifying the peculiar characteristics acquired in Uruguayan extensive grazing systems (low-cost production, higher tenderness, and healthy meat for human consumption), and without compromising animal welfare or the environment [8]. The objective of this experiment was, therefore, to evaluate the effect of the existing and emerging systems and temperament on animal welfare and on meat quality.

## 2. Materials and Methods

This study was run by the National Institute of Agricultural Research, at INIA La Estanzuela Research Station, Uruguay (latitude 34°20′18” south, longitude 57°41′24” west) over a period of 7 months. Eighty-four Hereford steers with a common origin and background on pasture (1.5 years old and 391 ± 24 kg of live weight) were randomly assigned to one of the following four treatments (T): T1, pasture (alfalfa (*Medicago sativa*), white clover (*Trifolium repens*), and fescue (*Festuca arundinacea*)) offered at 4% of live weight (LW); T2, pasture offered at 3% of LW and concentrate (corn 0.6% of LW); T3, pasture offered at 3% of LW and concentrate (corn 1.2% of LW); T4, concentrate (corn, sunflower pellets, and hay) ad libitum. Animals from T4 were allocated in three open-air plots on concrete flooring (9 m^2^ per animal). Animals from each treatment were divided in three pens of seven animals during the whole experiment (by electric fencing in pasture-based treatments and different plots in T4), for estimating pasture/concentrate ingestion and rejects. All animals had ad libitum access to water and mineral salts.

### 2.1. Field Determinations

#### 2.1.1. Productivity

Animals were weighed early in the morning without previous fasting every 14 days. Every 28 days, fat thickness and ribeye area were registered by ultrasound techniques.

#### 2.1.2. Temperament

Temperament was evaluated by both non-restrained and restrained tests using existing or adapted methodologies proposed by different authors [9,10,11,12,13]. Hair whorl position was recorded on the starting day, looking for correlations to temperament. If the center of the hair whorl was above the top of the eyes, the animal was categorized as “excitable”; it was categorized as “middle” if the center was located at eye level and “calm” if the center was located below the bottom of the eyes. Individual temperament was assessed once a month, after ultrasound measurements by three different tests. The first was crush score (CS), where the animal behavior is scored while it is in a chute, using a 1 (calm) to 5 (combative) scale, adapted from Hearnshaw [14] by Costa [15]. The categories took into account the general state of the animal including movements of limbs, head, and tail, as well as behavioral signs of stress, attributing one of the following scores: (1) animal does not offer resistance, remaining with tail, head, and relaxed ears; (2) animal has little limb movement, keeps head up and ears erect; (3) animal has frequent but not vigorous movements of limbs, head, ears, and tail; (4) animal offers great resistance, with sudden movements of head and tail, can jump and fall, with audible breathing; (5) paralyzed animal, with muscle tremor (freezing). The measurement was performed after the animal entered the chute. Only the rear (entrance) and front (exit) gates remained closed for the test, without the use of any of the containment structures (side walls, fisheries, and coasters). The records were taken 4 s after closing the gates. The second test was flight time (FT), where the amount of time (in seconds) it takes an animal to cover a known distance (5 m) immediately after leaving a confinement situation was recorded. A manual stopwatch was used, and registration started when the chute gate was opened, and the animal had the chance to exit. Animals with shorter exit times were considered more excitable. The third test was exit speed (ES), where data were obtained through a nominal scale scoring cattle exit gait of: 1 (walk), 2 (trot), and 3 (canter). A multicriterial temperament index (TIndex) was built from FT, CS, and ES, following Saaty [16], where a higher index indicated a calmer animal. The flight zone was registered twice for each treatment (the first day and 2 days before slaughter). The test was performed by the same person who slowly walked (2 steps/s) toward the group of animals, registering the distance (meters) when half of them turned away. Animals were in subgroups of 7; thus, an average value was calculated for each treatment.

#### 2.1.3. Physiology

Individual jugular blood samples were collected four times, once a month and also during bleeding at the abattoir. Blood samples were kept cool until they arrived at the laboratory when they were centrifuged at 3000 rpm for 15 min. The serum fractions were frozen and sent to Barcelona University (UAB), Spain, for acute phase protein (APP) concentration determination. APP-haptoglobin was measured in serum with a plate reader, ELISA (EMS Reader MF V2.9-0. Methodology: Bovine Haptoglobin ELISA test Kit, Life Diagnostics, Inc. Catalog number: 2410-7; Westchester, PA, USA). To prevent hemolysis effects, the methods of Hiss [17] were followed. Results are expressed in µg/mL.

Fresh fecal samples were taken directly from the rectum of all steers before each ultrasound measurement (four times, once a month). They were cooled for 15 min and immediately frozen and stored at −20 °C until sent to the University of Zaragoza, Spain, for cortisol/corticosteroid concentration determination. Fecal glucocorticoid metabolites (FGMs) were measured according to Morrow [18] using the commercially available I^125^ radioimmunoassay kit (Rats & Mice Corticosterone kit; ICN Pharmaceuticals). Samples were analyzed in duplicate. The inter- and intraassay coefficients of variation for fecal control samples were 4.3% and 6.7%, respectively. Results are expressed as nanograms of metabolite per gram of wet feces.

#### 2.1.4. Behavior

Cattle behavior was evaluated four times, once a month, in all treatments, by direct observation using the instantaneous scan sampling technique [19] within a 15 min sample interval. Observations were made from dawn to dusk (13 h a day) by four trained observers, who rotated 2 h between treatments, to minimize observer effect. At each scan, the following body postures and activities were recorded: lying or standing, walking, grazing, ruminating, drinking water, and others (any other non-standardized activity). As previously mentioned, animals from each treatment were divided in three pens of seven animals during the whole experiment. Each subgroup was considered the experimental unit within each treatment.

#### 2.1.5. Health Status

Any pathological event or trauma was observed daily, and the consequent medical treatments (if applicable) were registered, during the entire experimental period.

### 2.2. Postmortem Determinations

Animals were slaughtered in a commercial abattoir licensed for export following standards procedures for Animal Welfare, when they reached an average of 500 kg of LW in each treatment and at least 6 mm of fat covering (determined by ultrasound technique). Growth rates differed for all treatments and, consequently, concentrate-fed animals (T4) were slaughtered after 180 days, followed by steers from T3 after 197 days, from T2 after 225 days, and from T1 after 292 days of the experiment beginning. Good management practices were followed during all preslaughter stages (transport, loading, and unloading, and at the abattoir).

Carcass pH was measured at 24 h postmortem (pm) in the Longissimus thoracis (LT) muscle between the 12th and 13th rib using a pH meter (Orion 210A) with gel device. After 48 h, two steaks (2.54 cm thickness) were taken by our trained personnel at the abattoir between the 10th and 11th rib, vacuum-packaged individually, transported to INIA Tacuarembó Meat Laboratory, and aged for 7 and 20 days at 2–4 °C to determine tenderness (Warner Bratzler model D2000-WB). The LT steaks were placed inside polyethylene bags and cooked in a water bath until an internal temperature of 70 °C was achieved, using a Barnant 115 thermometer with type E thermocouple. Six 1.27 cm diameter cores were removed from each steak parallel to the muscle fiber orientation. A single peak shear force measurement was obtained for each core using the WB and an average value was calculated for each steak.

### 2.3. Statystical Analysis

Exploratory analyses were performed for all variables, using Statgraphics and SAS packages.

#### 2.3.1. Productivity

A general linear model (PROC GLM) [20] was used to evaluate the effect of treatment and temperament on productive variables. All interactions were considered and, when they were not significant, they were removed from the model.

#### 2.3.2. Temperament

Relationships between the initial and subsequent temperament measurements were analyzed in order to verify the consistency of each method. The three initial temperament appraisals were positively correlated with subsequent temperament assessments. On the basis of these exploratory analyses, a multicriterial temperament index (TIndex) was built from the FT, CS, and ES measurements. For that, a matrix was established with the relative importance of the FT, ATC, and CS characteristics to each other, according to our criteria. This matrix was normalized. A standardized ranking of the animals was generated for each of the variables, on a scale from 1 to 100. Then, the index was constructed according to the following equation: TIndex = $\overset{j}{\underset{1}{\sum }}W_{j}d_{i},$ where “W” is the weight of each of the variables according to the researcher’s criteria applying the analytic hierarchy process (AHP) [16] and “d” is the value of each normalized record. Considering that FT is an objective test, it was assigned a relatively higher ranking in the index, whereby a higher TIndex denoted a calmer animal. A mixed model with repeated measures was used to evaluate treatment effect on TIndex with time (four consecutive dates) with the animal as a random effect inside each treatment. A regression analysis was performed to study the effect of temperament on ADG.

#### 2.3.3. Physiology

Due to absence of normality, nonparametric tests (Mood’s and Kruskal–Wallis median test; PROC NPAR1WAY [20]) were performed to analyze the treatment effect on APP values at the farm and APP stress response at the abattoir. The same tests were applied to analyze the treatment and time effect on FGM. Regression models were used to analyze the relationship between physiological indicators and ADG.

#### 2.3.4. Behavior

A classification tree analysis was performed [21] to evaluate treatment effect on behavior frequencies. A logistic regression model was used to analyze daytime patterns of each observed behavior. According to grazing ethology in range cattle, a polynomic fourth-degree equation was fitted, and differences between treatments were analyzed. The logit model used was

Logit P_ijk_ = *β*_0_ + *β*_1_ DT_i_ + *β*_2_ DT_i_^2^ + *β*_3_ DT_i_^3^ + *β*_4_ DT_i_^4^ + T_j_ + ε_ijk_,

where Logit P_ij_ = log (p/1 − p) = log (response/absence of response) on each activity, DT_i_ = linear coefficient for daytime, DT_i_^2^ = quadratic coefficient for daytime (second-order), DT_i_^3^ = cubic coefficient for daytime (third-order), DT_i_^4^ = fourth-order coefficient for daytime (fourth-order), T_j_ = treatment effect, and **ε**_ijk_ = experimental error.

#### 2.3.5. Carcass and Meat Quality

Analyses of variance were carried out to study the effect of different factors and variables on tenderness, using initial and final live weight as covariates (PROC GLM) [20]. Regression analysis were also carried out to evaluate the effect of different variables on tenderness. Pearson correlation analyses were performed on data relating to production, physiology, and temperament. Means were compared using the LSMEANS procedure [20].

## 3. Results and Discussion

### 3.1. Field Determinations

#### 3.1.1. Productivity

Concentrate-fed animals (T4) had the highest average daily gain (ADG) which increased with the level of energy in the diet (Table 1). In general, when pastures and grains or concentrates are offered ad libitum, ADG is higher in concentrate-fed cattle, relative to grass-fed animals [22,23,24]. In our experiment, the use of grain even when not ad libitum (T2 and T3) increased ADG and decreased days to slaughtering. These differences were due to the total dry matter or digestive dry matter consumption, associated with different concentrate/energy proportion of the diet and probably with a lower energy expenditure for maintenance in T4. Fat thickness and ribeye area were also higher as long as supplementation increased (Table 1). Remaining pasture after grazing (more than 1.100 kg/dry matter) indicated that pasture consumption was not restricted in T1, T2, and T3 [25], being very relevant to AW. Assessing only one indicator of AW is unlikely to provide a complete understanding of the animal’s experiences [26]. Therefore, productivity is neither enough to ascertain an adequate AW status [27] nor to establish differences between production systems. However, it is relevant to welfare because it testifies that at least certain animal needs are being met.

#### 3.1.2. Temperament

In this experiment, hair whorl position was neither related to TIndex nor to productive variables. These results are not consistent with reports from Grandin [28], who indicated that cattle with a round hair whorl located above the eyes became significantly more agitated while they were restrained in a squeeze chute (crush) compared to cattle with a hair whorl located either between or below the eyes (*Bos taurus* and *Bos indicus* crossbreeds). With results similar to ours, and working with *Bos taurus*, Randle [29] reported that cattle response to humans in general was not significantly associated with hair whorl position.

**Figure 1:**
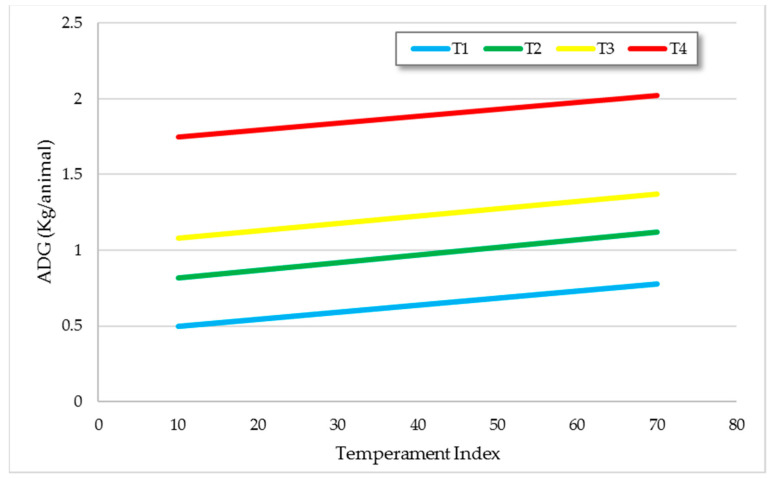
Average daily gains according to multicriterial temperament index (TIndex). Trendlines per treatment, estimated by regression analysis (*R*^2^ = 0.85).

None of the animals in our experiment were excitable or aggressive. However, TIndex had a significant effect on liveweight gains (ADG). Calmer animals had higher ADG within each treatment (*p* < 0.05; Figure 1). Voisinet [30] also reported a significant effect of temperament ranking on cattle. According to their results, *Bos taurus* steers with the calmest temperament had 0.19 kg/day greater (*p* < 0.05) average daily gain than steers with the most excitable temperaments. Several other authors reported that animals with calm temperament have better performance than excitable animals [30,31,32], whether in grazing [33,34,35] or in feedlot systems [30,33,36]. Animals that are fearful and move away from handlers are less productive [37,38], and reduced growth is considered to be the consequence of a series of acute or chronic responses to human presence [39,40].

**Figure 2:**
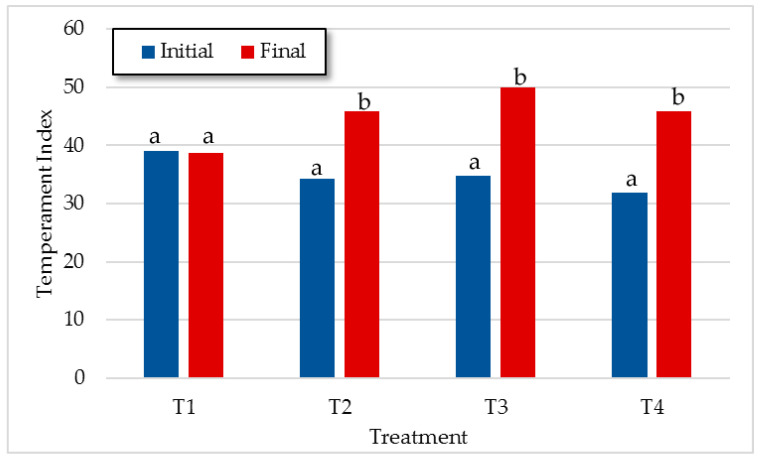
Least square means for initial and final TIndex within treatments. ^a,b^ Means within the same treatment with different letters differ with *p* < 0.05.

**Figure 3:**
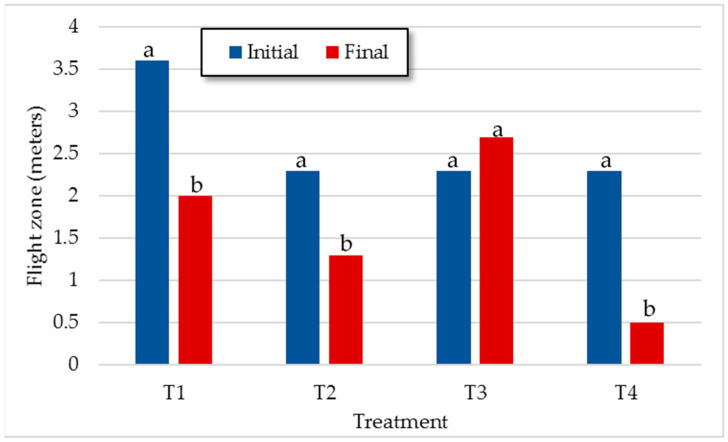
Mean values of initial and final flight zone for each treatment. ^a, b^ Means within the same treatment with different letters differ with *p* < 0.05.

Figure 2 shows the initial and last TIndex comparison within each treatment. It was observed that animals from T2, T3, and T4, where human contact was more frequent, became calmer as the experiment progressed. Although all animals were used to handling and penning because of frequent weighing and a strict protocol of good management practices was followed during the whole process, steers from T1 had less daily contact with humans. However, from flight zone determinations (Figure 3), we could say that, at the group level, animals from T1 also improved their reaction to humans. Therefore, according to our results, animal temperament and human handling are key factors for minimizing stress. Mays [41] and Ceballos [42] also reported changes in animal temperament when periodic handling is applied. According to Grandin [43], when no painful procedures are performed, with the exception of vaccination or blood collection, the temperament scores decreases as animals become calmer with repeated handling and restraint sessions.

The flight zone (FZ) is the animal’s personal space, and its size depends on the tameness of livestock. When someone enters its space, the animal will move away. It is clear that all animals in this study were tame and were habituated to human proximity (the higher final FZ was 2.5 m in T3; Figure 3). The feedlot steers showed the greatest difference between initial and final values of FZ at the end of the experiment (Figure 3).

Similar results were found by Uetake [44] who reported that the FZ of cows was gradually reduced throughout the experiment. It is more than likely that the good management practices followed in our experiment had an important effect on FZ values. These results suggest that the stockperson and handling procedures could be even more relevant than the rearing system in determining the FZ.

#### 3.1.3. Physiology

**Figure 4:**
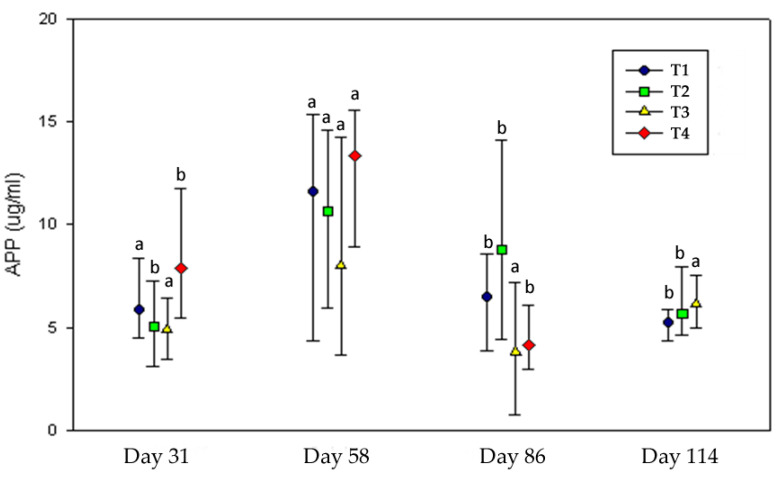
Acute phase protein (APP) concentration in the different treatments throughout the experiment. Lines represent the median, 25th, and 75th percentile values. ^a,b^ Medians within the same treatment at different dates differ with *p* < 0.05. Animals from T4 were already slaughtered at day 114.

Bovine Haptoglobin (APP)—The acute phase proteins (APP) are a group of blood proteins whose concentration changes in animals subjected to external or internal challenges including stress; as such, APP analysis may be used to help monitor the health and welfare of production animals on the farm [45] or to identify the disease and health status of animals at slaughter. Changes in their concentrations occur early in the course of disease before manifestation of clinical signs. Therefore, they could be also used as an early indicator in various pathological processes [46]. In this experiment, APP concentration did not differ between treatments within each date (*p* > 0.05) because of a high variability (see 25th and 75th percentile values in Figure 4), but it was significantly affected by the time period (Figure 4). Results from the Kruskal–Wallis and Mood median tests showed that treatment 2 and treatment 4 had a significant rise in APP concentration at day 58 (Figure 4). Animals suffering illness were not considered in this analysis, but subclinical states of disease or inflammation processes not detected by us could have contributed to these results. Nevertheless, APP was merely interpreted as a stress response probably due to environmental conditions (confinement, diet, weather). Similar results were found by Arthington [47] when comparing stress in early and normal weaned calves by APP concentration. Although no calf became sick, these authors reported differences in APP concentration between treatments attributable to a stress response and also reported an inhibition of animal growth. Significant evidence exists that APP can directly inhibit animal growth [48]. However, in the present experiment, APP stress response was not enough to induce variations in live weight gains (*p* > 0.05). Although the mechanism of APP induction in response to stress has yet to be elucidated, activation of the hypothalamic–pituitary–adrenal (HPA) axis by stress signals may be a trigger of systemic or local (intra-pituitary) cytokine production, thereby augmenting hepatic APP synthesis and release into the bloodstream [49]. In this study, temperament was not related to the APP stress response (*p* > 0.05).

**Figure 5:**
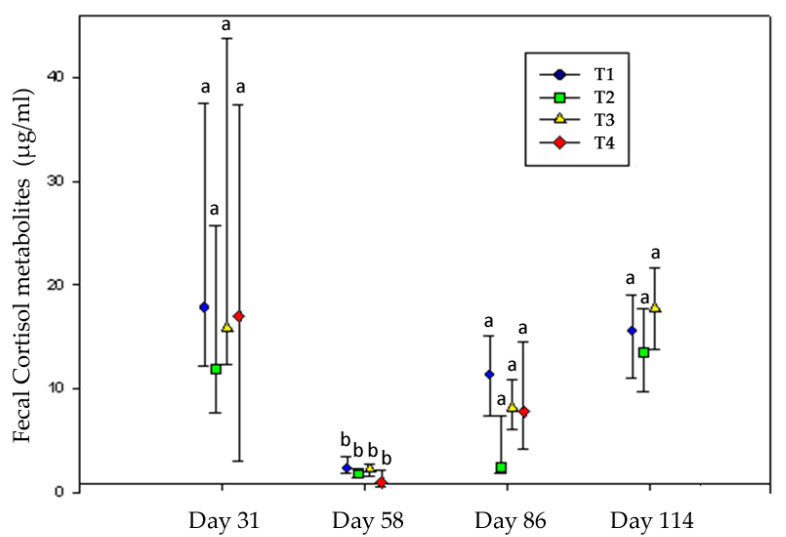
Fecal cortisol concentration in the different treatments throughout the experiment. Lines represent the median, 25th, and 75th percentile values. ^a,b^ Medians within the same treatment at different dates differ with *p* < 0.05. Animals from T4 were already slaughtered at day 114.

Fecal Glucocorticoid (FGM)—Stimulation of the sympatho-adrenal and hypothalamo-adrenal systems results in the production of catecholamine and corticosteroid hormones, being primarily an adaptive mechanism that allows the animal to cope with the stressor by mobilizing body reserves and regulating inflammatory response to injury [18]. However, sustained or long-term increases in the hypothalamic–pituitary–adrenal axis (HPA) activity are thought to be detrimental [50,51]. Cortisol, as a major stress hormone, is a benchmark measurement of stress responses in humans and animals [52]. Fecal glucocorticoid metabolite (FGM) assays reflect the average level over a time period rather than a point sample and, therefore, may provide a more accurate assessment of long-term glucocorticoid levels [53]. In ruminants, the concentration of cortisol metabolites in a fecal sample reflects cortisol production about 12 h earlier [54]. Being a noninvasive endocrine indicator, it has become an increasingly popular method of animal welfare assessment both in production and in wildlife species. In this experiment, FGM concentration did not differ between treatments within each date; however, just like APP, it was significantly affected by time (*p* < 0.05). Results from the Kruskal–Wallis and Mood median tests showed that all treatments had a significant decrease in FGM values at day 58, during the second month of the experiment (Figure 5).

Animals from all treatments were probably suffering from long-term stress and were trying to cope with a potent stressor, which may have suppressed adrenocortical responses. The day previous to the FGM determination, heavy rainfall was registered (40 mm) [55]. Considering that this winter was particularly dry and combined with a high wind registered that day (113 km/h), the precipitation could have contributed to the long-term stress. According to Salvin [56], animal comfort and productivity can also be compromised during exposure to cold, wet, or windy conditions, as there is an increase in maintenance energy requirements [57,58]. We, therefore, considered that, due to this potent stressor, the activity of the system was probably changed due to a chronic stress state which reduced the sensitivity of the HPA axis. Under these circumstances, each level of the axis is subjected to opposing influences (stimulation and feedback through corticosteroid hormones). This resetting of the HPA axis at a different level of activity was probably the state of resistance [59]. Similar results were found by Fisher [60] when subjecting finishing heifers to different space allowances.

An increase in glucocorticoid secretion does not automatically equate to a state of distress [61,62], but the chronic activation of the HPA axis observed in the present experiment suggests a high level of stress and probably involved suffering in all treatments at this moment. Animals from all treatments were able to recover normal activity of the HPA axis on the following date (Day 86, Figure 5). It seems that adaptation to environmental challenges at the level of the pituitary–adrenocortical system are flexible and dynamic over time. They may involve sensitization and desensitization, for example, due to reversible changes in the density and sensitivity of adrenocorticotropic hormone and cortisol receptors [63].

It is important to mention that HPA activation in response to an aversive event depends on the control the animal can exercise on this event [64]. Animals from T4 were space-restricted and were forced to be in permanent social contact; more importantly, they had fewer opportunities to engage in behaviors that counteract the negative effects of the stressful event. Moreover, they were probably under a subclinical acidosis state because of the high level of concentrate in the diet. All these conditions, considering the simultaneous rise in APP, could have made the process more difficult and, consequently, would have induced a higher stress response in T4. However, the former did not result in a significant biological cost that shifted energy away from normal processes [61], with no significative effect of FGM on ADG in any treatment, whether during this period or throughout the whole experiment. In this study, temperament was not related to the FGM stress response (*p* > 0.05).

**Figure 6:**
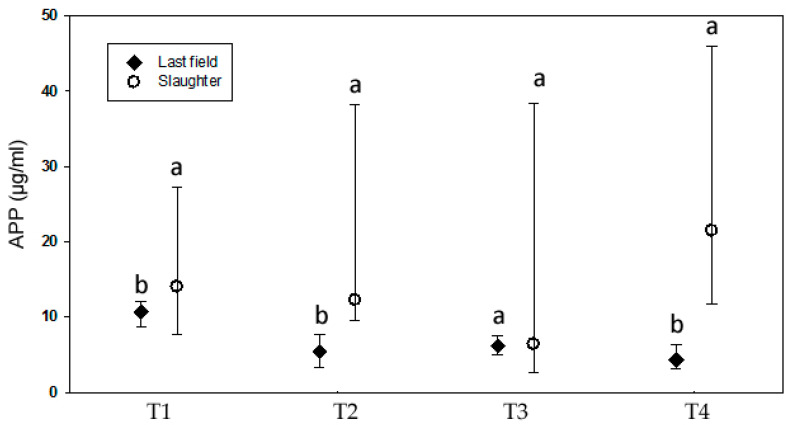
Last field and slaughter APP serum concentration within each treatment. Lines represent the median, 25th, and 75th percentile values. ^a,b^ Medians within the same treatment with different letters differ with *p* < 0.05.

APP pre slaughter—The preslaughter stress response was also studied through APP concentration in the blood. In T1, T2, and T4, APP values at slaughter (debleeding) were higher than preloading determinations (*p* < 0.05; Figure 6). In T3, differences were not significant, but a high variability was observed in APP at slaughter (Figure 6).

From the previous results, it is evident that the preslaughter stage is very stressful for cattle regardless of the fattening system. Animal handling at slaughter plants has the potential to be rough; not only does this negatively impact meat quality, but it can also compromise animal welfare [65]. Moreover, isolation from herd or flock mates is often highly stressful to both sheep and cattle. In this case, isolation occurred at the stunning box prior to slaughter. Isolated single cattle may respond with agitated behavior, increased physiological indicators of stress, or increased vocalization [66]. If the stress response is strong enough, production and meat quality will be affected [65], causing major economic losses to the industry [67]. However, in our experiment, this stress response was not enough to negatively affect MQ (see shear force discussion).

In the present experiment, individual temperament had a significant effect on preslaughter stress (*p* < 0.05). As reported by Bourguet [68], inherited differences in fearfulness or reaction to isolation may affect the intensity of an animal’s reaction. This highlights the importance of proper animal handling throughout their entire lives, but mainly in the stages prior to slaughter, where animals are specially challenged by novelty and stress-inducing situations. By identifying problem areas and behaviors, resources can be utilized to provide better training, and increasing third party audits can help reduce the problem [65].

#### 3.1.4. Behavior

**Figure 7:**
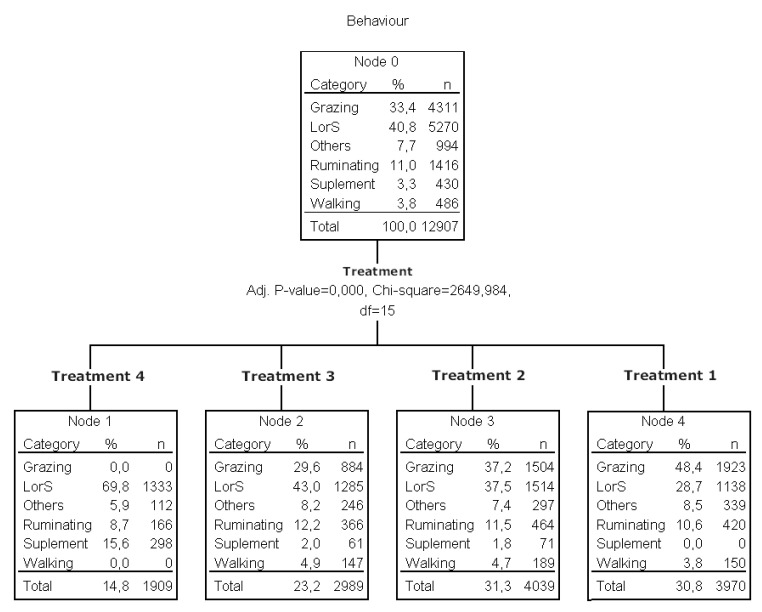
Treatment classification based on behavior frequency distribution. LorS: lying or standing.

Cattle normally have daily alternate periods of grazing, ruminating, and resting [69]. The duration and distribution of these behaviors are influenced by nutritional characteristics, management, climatic conditions, and animal activity in the group [70,71]. Primarily based on standard patterns, behavior has also emerged as a focal point for welfare deliberation and it could be highly related to feelings [72]. Positive experiences such as comfort, play, exploration, and contentment should be considered as criteria for welfare [73], especially when comparing different production systems. In this experiment, on the basis of behavior frequencies, all treatments were discriminated when using a classification tree multivariate procedure [21] (Figure 7), and differences in time allocated to each behavior are shown in Table 2.

**Figure 8:**
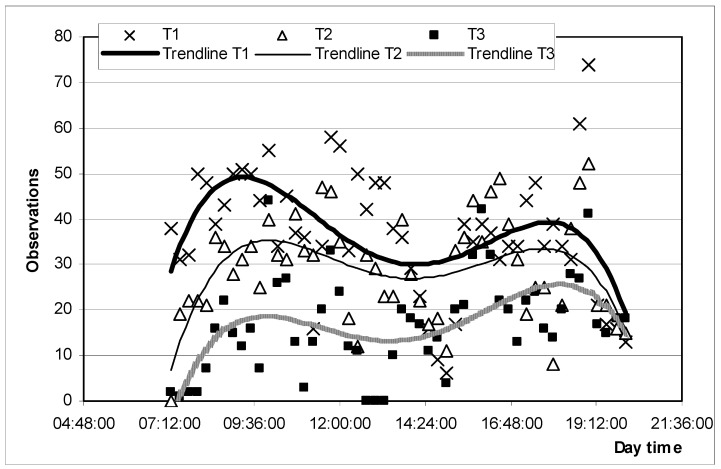
Daytime grazing frequency and distribution by treatment.

Grazing. Animals from T1 spent more time of the day grazing when compared to T2 and T3 (Figure 7, Table 2). Similar results were found by Wagnon [74] who showed that daily feeding of supplements disrupted normal grazing activity and reduced daily grazing time in range cows. Grazing time in cattle is between 4 and 14 h per day, with most observations between 7 and 11 h [69]. According to Poppi [75], if grazing time is more than 12–13 h per day, ingestive behavior components could be affected. Hodgson [76] considers that grazing time higher than 9 h might indicate some kind of pasture restriction. In our experiment, grazing time was 6 h in T1, 4.5 h in T2, and 3.6 h in T3, but these differences in time spent grazing were not considered relevant to animal welfare, especially considering the high quality and availability of the pasture. In addition, we did not register night grazing behavior, which was probably important. Although grazing behavior is affected by various environmental conditions [77], most grazing behavior studies show similarity in daily grazing patterns, with the major grazing period occurring early in the morning and another later in the afternoon, with intermittent grazing occurring throughout other periods of the day and night (baseline ethogram) [78]. In our experiment, periodical grazing patterns were not altered due to the supplementation effect (Figure 8). One criterion of good animal welfare is the expression of a time budget similar to that expressed by free-ranging conspecifics [79].

Grazing was obviously deprived in T4. According to de Pasillé [80], the performance of a behavior associated with feeding can directly influence the digestive processes even if the consumption of food and nutrient intake is not altered. Therefore, deprivation of grazing in T4 could be affecting animal welfare in advance.

**Figure 9:**
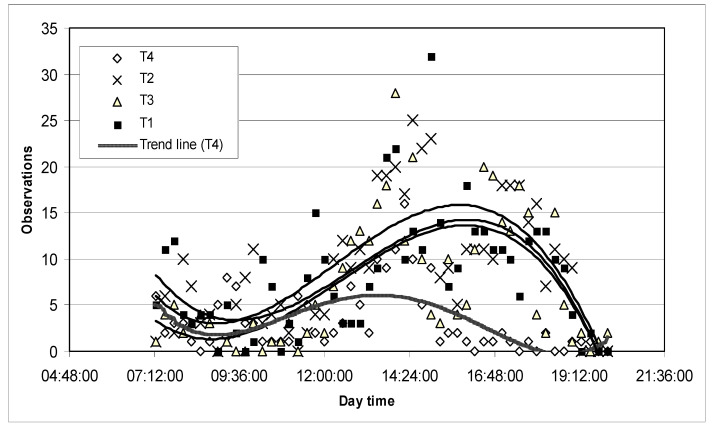
Ruminating frequency and distribution during daytime by treatment.

Ruminating. Time spent ruminating is a direct indicator of animal welfare. In this experiment, it was similar for steers in pasture-based treatments: T1, T2, and T3 (Figure 7, Table 2). Similar results were found by Adams [81], reporting no differences in ruminating time between pasture-based cows, both supplemented and non-supplemented. As ruminating time may change due to cell-wall material consumed (associated with pasture ingestion), these results suggested that the supplement did not substitute pasture ingestion, being positive from the AW perspective. Time spent ruminating was lower in T4 than in other treatments (Table 2). Although hay was ad libitum in T4, pasture-based treatments ingested more cell-wall material and, consequently, spent more time ruminating. Ruminating time and number of ruminating bouts are likely to affect overall production, because of buffering the rumen with copious amounts of saliva, which enhances the rumen environment [82]. In this experiment, the reduction in time ruminating did not compromise overall production in T4, but individual problems emerged, probably due to this factor among others (two animals showed dietary diseases and one of them died). Periodical patterns of rumination are shown in Figure 9, with no differences among T1, T2, and T3. Ruminating time could not be compared in absolute terms, because animals were not observed during the night, when they are thought to perform the highest frequency of this behavior [76,83].

Lying or standing. Animals from T3 spent more time lying or standing, with intermediate values for T2 and lower values for T1. Animals from T4 spent more time than pasture-based treatments lying or standing (LorS; Figure 7). However, as confined animals had fewer choices available and several behavior patterns were suppressed, it would be a subjective criterion to categorize these behaviors as “resting” in T4. These results could probably mainly be explained by their spatial restrictions.

Walking. Walking frequencies were not different in T3, T2, and T1 and were all higher than T4 (*p* < 0.05; Figure 7). Walking is associated with exploration. Regarding exploration, it is also reported that cattle give a very high priority to this behavior [84] and it may be included in the “behavioral needs” category. In addition to being deprived of grazing in T4, the restriction of other relevant behaviors, such as walking, exploration, and ruminating, probably affected animal welfare in that treatment, making it necessary to investigate the motivational systems underlying each separate behavior to reach firm conclusions about the influence of behavioral restriction on animal welfare [85]. In addition, animals from T4 may not have met their individual space needs, they may not have established spatial distribution according to social structure, they had a more uncomfortable lying surface (no bedding area), they may not have exercised, rested, or roamed, and they may have suffered more from fear when threatened. It is important to mention that they were unable to escape or to adequately face situations such as bad weather conditions like the event presented on Day 58 (Figure 5). All these factors would have contributed to a higher level of boredom, frustration, depression, or despair from an unrewarding environment and, consequently, suffering in T4. Future studies should evaluate pathological and aggressive behavior as indicators of discomfort, boredom, or frustration. The new concept of animal welfare not only includes the possibility of expressing natural behavior, but also the absence of negative emotional states [67]. Even further, Boissy [86] reported that animal welfare scientists are becoming increasingly aware of the importance of both positive and negative affective states. According to Salvin [56], there is a perception that housing cattle in feedlots may negatively influence affective states due to the inability to express natural levels of activity, such as would occur during grazing, potentially resulting in “boredom” [87]. Therefore, we agree with the previous authors in relation to the importance of current animal welfare assessment systems taking into account these new animal needs and including appropriate indicators (valid, reliable, and viable) that allow assessing both the physical and the mental welfare of the animals.

Supplementing frequencies were not different in T3 and T2. Regarding time spent eating concentrate, T4 was higher than T3 and T2, as expected.

#### 3.1.5. Health Status

Health status in T1, T2, and T3 was satisfactory without differences between treatments, and no specific medical treatments were required throughout the experiment. Although preventive measures were applied and health monitoring was permanent, as previously mentioned, two animals showed dietary diseases in T4 and one of them died because of tympanism (4.8% mortality rate). Health status is an important welfare indicator in farm animals, and, regardless of the rearing system, it must be a fundamental target for animal’s quality of life [88]. Mortality is also a clear measure of poor welfare, not only because animals that die have obviously failed to cope, but also because high losses in a given environment show that even those individuals that do not die may have serious difficulties in coping [89]. In this experiment, transition was done according to the same strategy for treatments including the use of concentrate (T2, T3, and T4). An accustoming period of 15 days (with incremental supplies of concentrate) was carried out, and they were constantly monitored during the experiment. However, we consider that the strategy for preparing the animals in T4 should have been stricter than that for T2 and T3, in relation to both the food transition and the greatest challenges they would have to face, regarding spatial distribution and social structure. We also consider that each individual and their health history prior to entering the feed lot could have also contributed to these problems in T4. According to Salvin [56], the lifetime health history of cattle may influence welfare within feedlots. Diagnosis and treatment of illnesses both before and after feedlot entry may have a lasting influence on the health status and overall performance of individual animals while in the feedlot [90]. Even considering animals from the present experiment were directly purchased from one owner who keeps good health records, we did not have such information. Identifying and treating sick or injured animals, as well as understanding their health history before feedlot entry, are important steps for improving not only the welfare but also the performance of feedlot cattle [56].

We previously saw that animals from the feedlot system had the highest ADG and final live weights. From a different perspective, we can now say that those animals could have experienced higher stress throughout the experiment due to the deprivation of grazing, spatial restrictions, and the daily pattern alteration of other relevant behaviors. In addition, their health was compromised, and mortality was present. This reinforces the theory that assessing only one indicator of animal welfare is unlikely to provide a complete understanding of the animal’s experiences [26], and productivity is definitively not enough to assess welfare. On the contrary, as in our experiment and in agreement with Rollin [72], profitable production systems may be the cause of certain production problems and diseases.

### 3.2. Postmortem Determinations

pH values at 24 h pm were higher in carcasses from T1 than in those from T2 and T4 (Table 3). According to Immonen [91], glycogen levels in muscle increase with metabolizable energy intake, and the effect of a high-energy diet on the muscle content was reflected all the way to ultimate pH values. In addition, Muir [92] suggested that animals from pasture diets are more susceptible to preslaughter stress; thus, they could have less glycogen content in muscle and, consequently, higher final and intermediate pH values than grain-fed steers. In spite of the differences in initial and final pH values in our experiment, all were within the normal range (5.5–5.8; Table 3). Moreover, shear force values (WBSF) with 7 and 20 aging days, were higher in concentrate-fed beef than in meat from forage-fed cattle (Table 3), being consistent with Realini [93], who reported 2.8 and 3.5 kg (WBSF) for pasture- and concentrate-fed animals, respectively, after 14 aging days. In our experiment, pH values closer to neutrality in T1 could have a positive effect on calpain activity and, consequently, on tenderness. However, previous results from Uruguay [93] showed that shear force is lower in meat from forage-fed cattle than in concentrate-fed beef, even without pH differences (pH 5.7 ± 0.039 in pasture and 5.7 ± 0.028 in concentrate). These tenderness results are consistently and repeatedly being reported in Uruguayan and in Argentinian conditions, and they could probably be explained by certain effects in the pre- and postmortem phase (such as proteolysis, glycolysis, and ATP turnover related to preslaughter stress), but mainly by other factors that are currently being deeply discussed in Uruguay. Among these other factors, we highlight connective tissue (related to skeletal maturity) and marbling.

Table 3 shows that WBSF decreased in all treatments with aging time.

**Figure 10:**
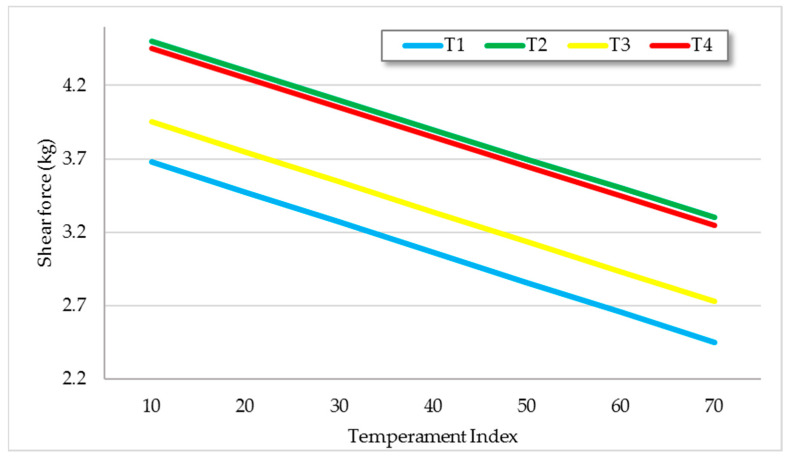
Shear force (kgF) with 20 aging days according to TIndex. Trendlines per treatment, estimated by regression analysis (*R*^2^ = 0.30).

Temperament had a significant effect on WBSF after 20 aging days (*p* < 0.05). Calmer animals had lower WBSF values in all treatments. The interaction between treatment and TIndex was not significant, such that slopes were parallel implying that WBSF decreased at the same rate in all systems as TIndex increased (Figure 10).

Our results are consistent with those reported by Olson [94], reporting that loin steaks from calm heifers had 8% lower (<0.05) WBSF than steaks from excitable heifers. Other authors such as Fordyce [95] also established that cattle with wilder temperament produce tougher meat [96]. Animals with the most excitable temperament may be most susceptible to stress generated by routine handling practices, such as loading and unloading, transport, and the new environment in the abattoir [97]. This stress is likely to reduce muscle glycogen level in vivo [98] because of energy expenditure due to physical exercise or psychological stress, which may in turn increase the ultimate pH of muscles [99] and have a significant effect on meat shear force. In the present experiment, there were no excitable animals and pH values were within normality; however, as reaction to stress varies according to individuals, it is conceivable that additional stressors near the time of slaughter would have provided sufficient stress levels in moderately stress susceptible animals [96].

Considering its commercial importance and consumer satisfaction according to WBSF [100], tenderness was divided into three categories in order to establish relationships with temperament (Table 4).

With 7 aging days, beef with shear force values less than 3 (which means 100% consumer acceptance) came from calmer animals. Beef with WBSF values from 3 to 4 came from animals with an intermediate TIndex, and the least tender beef came from the more excitable animals (Table 4). This effect was still relevant with 20 aging days. According to Voisinet [96], a possible hypothesis for temperament effects on tenderness may involve adrenaline action on *β*-adrenoceptors. Adrenaline binding at *β*-adrenergic receptors on muscle cell membranes mediates glycogen metabolism. *β*-Adrenoceptor agonists have been implicated in increasing shear force values [101]. From this, it is possible to postulate that a relationship between *β*-adrenoceptor density or affinity and temperament could possibly explain a portion of the effects on meat quality [96]. The correlation coefficient between TIndex and pH at 24 h postmortem partially confirms this hypothesis (*r* = −0.53; *p* value = 0.01). In addition, the altered metabolism associated with greater stress responsiveness in the more stressed cattle may have created conditions that were less favorable to calpain-mediated proteolysis [102]. A high rate of protein turnover in the muscle at the time of slaughter is important to improve tenderization, and, provided pH is low, such animals will become acceptably tender.

According to the results of this experiment, production systems based on pastures that ensure an adequate level of nutrition and health (T1, T2, and T3) would be more appropriate for fattening animals, from the animal welfare perspective. This, associated with the image that exists that they are more natural or healthy, would allow a differentiation of these products in the international market. Added to this, the pasture treatment showed greater meat tenderness. Intensification up to certain levels and without deprivation of certain behaviors (T2 and T3) should provide productive benefits without compromising animal welfare. Feedlot systems (T4) with 9 m^2^ per animal would compromise animal welfare because of the negative effect on relevant behaviors and health. It is considered that, if some conditions of the confined system are improved, such as the space available per animal, the adjustment of the transition strategy, and a strict prevention and constant monitoring of diet disorders, this alternative could be valid for fattening cattle.

## 4. Conclusions

Individual productivity increased with the level of energy in the diet. Temperament had a significant effect on live weight gains within each treatment, and frequent and proper handling improved animal reactions to regular handling procedures. Rainfall combined with cold and wind appeared to be a potent stressor for cattle, with no incidence on production indicators. The integration of different types of indicators is essential when drawing conclusions on animal welfare. In the present experimental conditions, animal welfare was negatively affected in T4, being evident through physiological indicators, the restriction or deprivation of relevant behaviors, diet-related diseases, and mortality. Immediate preslaughter stress seems to be very important for cattle, highlighting the relevance of a permanent proper handling, especially at this stage. Therefore, specific studies should be undertaken for minimizing it. Regardless of the feeding strategy and even considering that animals were calm, temperament appears to be an important factor, considering its influence on productivity, on the preslaughter stress response, and on meat tenderness. Meat tenderness was highest in T1. Research should also be conducted regarding differences in the tenderization biochemistry process associated with feeding strategies, temperament, and preslaughter stress.

## Figures and Tables

**Table 1:** Average daily gain, ribeye area gain, and fat thickness gain by treatment. Least square means ± standard error.

	Treatment			
	T1	T2	T3	T4
Average daily gain (kg/an)	0.52 ^d^ ± 0.05	0.94 ^c^ ± 0.05	1.12 ^b^ ± 0.05	1.56 ^a^ ± 0.05
Ribeye area gain (cm^2^/day)	0.05 ^b^ ± 0.01	0.08 ^b^ ± 0.01	0.08 ^b^ ± 0.01	0.17 ^a^ ± 0.01
Fat thickness gain (mm/day)	0.03 ^c^ ± 0.00	0.04 ^b^ ± 0.00	0.06 ^a^ ± 0.00	0.07 ^a^ ± 0.00

^a,b,c,d^ Means within the same line with different letters differ with *p* < 0.05.

**Table 2:** Time budget associated with each behavior (differences among treatments).

	Time Budget—Difference among Treatments (*p* < 0.05)
Grazing	T1 > T2 > T3
Ruminating	(T1 = T2 = T3) > T4
Supplementing	(T2 = T3) < T4
Lying or standing	T1 < T2 < T3 < T4

**Table 3:** pH at 24 h postmortem and Warner Bratzler shear force (WBSF) (kgF) after 7 and 20 aging days by treatment. Least square means ± standard error.

	Treatment			
	1	2	3	4
pH 24 h	5.70 ^a^ ± 0.03	5.54 ^b^ ± 0.03	5.73 ^a^ ± 0.03	5.47 ^b^ ± 0.03
WBSF 7 days	3.17 ^c^ ± 0.25	4.17 ^ab^ ± 0.25	3.57 ^bc^ ± 0.24	4.51 ^a^ ± 0.30
WBSF 20 days	2.87 ^b^ ± 0.16	3.78 ^a^ ± 0.16	3.26 ^b^ ± 0.16	3.98 ^a^ ± 0.19

Values with different letter in the same line differ with *p* < 0.05. WBSF: Warner Bratzler shear force.

**Table 4:** Temperament index according to WBSF (kgF) by category. Least square means.

	WBSF < 3 (100% CS)	3 > WBSF < 4 (99% CS)	WBSF > 4 (86% CS)
7 aging days	45 ^a^	36 ^ab^	26 ^b^
20 aging days	40 ^a^	35 ^ab^	22 ^b^

^a,b^ Means within the same line with different letter differ with *p* < 0.05. WBSF: Warner Bratzler shear force, CS: consumer satisfaction.
